# Prevalence and Predictors of Immunological Failure among HIV Patients on HAART in Southern Ethiopia

**DOI:** 10.1371/journal.pone.0125826

**Published:** 2015-05-11

**Authors:** Kesetebirhan Delele Yirdaw, Susan Hattingh

**Affiliations:** 1 Department of Health Studies, University of South Africa, Addis Ababa, Ethiopia; 2 Dean Office, College of Nursing Al-Ahsa, King Saud bin Abdulaziz University for Health Sciences (KSAU-HS) National Guard Health Affairs, Eastern Region, Saudi Arabia; University of Athens, Medical School, GREECE

## Abstract

Immunological monitoring is part of the standard of care for patients on antiretroviral treatment. Yet, little is known about the routine implementation of immunological laboratory monitoring and utilization in clinical care in Ethiopia. This study assessed the pattern of immunological monitoring, immunological response, level of immunological treatment failure and factors related to it among patients on antiretroviral therapy in selected hospitals in southern Ethiopia. A retrospective longitudinal analytic study was conducted using documents of patients started on antiretroviral therapy. Adequacy of timely immunological monitoring was assessed every six months the first year and every one year thereafter. Immunological response was assessed every six months at cohort level. Immunological failure was based on the criteria: fall of follow-up CD4 cell count to baseline (or below), or CD4 levels persisting below 100 cells/mm^3^, or 50% fall from on-treatment peak value. A total of 1,321 documents of patients reviewed revealed timely immunological monitoring were inadequate. There was adequate immunological response, with pediatric patients, females, those with less advanced illness (baseline WHO Stage I or II) and those with higher baseline CD4 cell count found to have better immunological recovery. Thirty-nine patients (3%) were not evaluated for immunological failure because they had frequent treatment interruption. Despite overall adequate immunological response at group level, the prevalence of those who ever experienced immunological failure was 17.6% (n=226), while after subsequent re-evaluation it dropped to 11.5% (n=147). Having WHO Stage III/IV of the disease or a higher CD4 cell count at baseline was identified as a risk for immunological failure. Few patients with confirmed failure were switched to second line therapy. These findings highlight the magnitude of the problem of immunological failure and the gap in management. Prioritizing care for high risk patients may help in effective utilization of meager resources.

## Introduction

In Ethiopia, access to highly active antiretroviral therapy (HAART) started on massive scale in 2005 and by 2013 it has reached to some 317,443 people [1, 2]. Treatment has been successful as demonstrated by the improvement of survival in many settings in Ethiopia [3, 4]. This has changed the course of the epidemic and made Human Immunodeficiency virus (HIV) a chronic manageable disease.

As important as it is to get people who need the treatment be on HAART, it is even more important to ensure that patients adhere to the treatment so that they benefit from it in the long run as well. This is because poor adherence to treatment is associated with increased risk of drug resistance and thus increased risk to treatment failure [5, 6]. Achieving adherence to treatment of ≥95% is not always possible for all people on HAART because of for example lack of family support, unemployment, alcohol intake, poverty and the presence of other conditions and diseases that could limit insight and knowledge [7]. In Ethiopia, studies indicate that the level of adherence is suboptimal for patients on HAART ranging from 7–28% based on self-report and/or pill count [7–13]. The longer a person is on a failing HAART regimen, the higher the mortality risk [14]. For this reason, monitoring HAART treatment response, especially immunological and virologic, which is challenging issue in resource limited chronic HIV care settings, becomes very important. Detection and management of treatment failure could be compromised if the monitoring is not carried out timely [15, 16]. For resource limited settings, the World Health Organization (WHO) recommends clinical and immunological monitoring of treatment with targeted viral load testing when available [17]. In Ethiopia viral load testing is not available in routine practice settings; hence immunological monitoring is the only option for laboratory based monitoring.

Literature is scanty on the issue of immunological monitoring in Ethiopia, but the available data reveals a suboptimal CD4 monitoring among pediatric patients on HAART in southern Ethiopia with only 68 and 37% of children having 6^th^ and 24^th^ month follow-up CD4 tests (n = 1163) [18]. As for immunological response after HAART, studies in Ethiopian patients on HAART report CD4 increment to be the greatest in the first six to twelve months with a pattern of slight decrease in mean CD4 count in later years of follow-up [4, 19, 20]. While the pattern of CD4 response is similar to what is reported in other African countries, it says little about those with immunological failure. In Ethiopia, only two studies done estimated the prevalence of immunological failure among pediatric patients, which was around 10% [21, 22] and a single study assessed it among adults and the prevalence of failure was 22% [23]. Another study found out the prevalence of those on second line HAART in multiple hospitals and health centers in Ethiopia to be 2% at 24 months, a much lower figure than the reported suboptimal adherence, which may be a reflection of low immunological monitoring and/or failure of detection by health care workers [15, 16, 24].

There is no published study for adult patients in Ethiopia indicating the level of adherence to immunological monitoring guidelines, which by far accounts for more than 90% of patients on HAART. Furthermore, there is no study indicating the prevalence and determinants of patients with immunological failure that may benefit from targeted viral load monitoring in the Ethiopian care setting. This study, therefore, investigated the level of immunological monitoring, the level of immunological response, both in the form of CD4 cell counts, as well as the prevalence of treatment failure and its determinants in selected areas in Ethiopia with a focus on immunological criteria.

## Methods

### Ethical Issues

Approval for this study was obtained from the National Research Ethics Review Committee, and the Southern Nations Nationalities and People’s regional health bureau of Ethiopia. Ethical approval was also sought from the Department of Higher Degrees of the Department of Health Studies at the University of South Africa (UNISA). Permission was also obtained from the directors of each of the hospitals from whose HIV clinics data was used for the purpose of this study. Informed consent was not obtained since there was no interaction with human subjects. In order to keep anonymity, data was de-identified (de-linked) before analysis.

### Study Design and Setting

This is a retrospective, longitudinal study conducted in SNNP region, which is located to south of the Ethiopian capital. It has a population of around 18 million people [25]. According to the latest estimates, the HIV prevalence in the region is 0.7 percent in 2014 [26]. There are 22 public hospitals spread across 17 zones/special woredas providing HAART since 2005. Two Hospitals were selected by convenience sampling for the study. Each facility has HAART clinic with at least one trained clinician who takes care of clinical management of patients, one data clerk who ensures that medical records are standardized, registers are updated, and electronic database is updated every day, and one case manager and two adherence supporters to trace clients who miss their appointment or are lost to follow-up from care. In each facility, a CD4 cell count machine is available. During the study time, people living with HIV (PLHIV) fulfilling the following criteria were eligible for HAART: all WHO stage IV clients, WHO stage III clients with CD4≤350 and WHO stage I or II with CD4≤200. In the absence of CD4 testing, patients with WHO stages III or IV would be eligible for HAART and CD4 would be determined as soon as it was available after treatment initiation. Later on the CD4 based eligibility was modified to include all those with CD4≤350 no matter what the WHO stage was. The first line ARV regimens used were stavudine (d4t) or tenofovir (TDF) or zidovudine (AZT) plus lamivudine (3TC) plus nevirapine (NVP) or efavirenz (EFV). [27]

### Immunological Monitoring and Immunological Treatment Failure Criteria

Immunological monitoring (CD4 cell count test) is expected at baseline just before starting HAART. After starting treatment CD4 cell count should be measured every six months for the first year, then once yearly in line with the health management information system (HMIS) guidance for cohort reporting [28].

Although the WHO recommends three types of criteria to define antiretroviral treatment failure, namely clinical, immunological, and virologic, the focus of this study was on immunological criteria which the WHO defines as follows:

- fall of follow-up CD4 count to baseline (or below), or
- CD4 levels persisting below 100 cells/mm^3^, or
- 50% fall from on-treatment peak value.

These same criteria were used in this study to identify patients with immunological failure according to the WHO 2010 guidance [17]. A patient must be followed for a minimum of six months before assessment is made for treatment failure.

### Study Period and Population

PLHIV five years and older started on HAART in the selected facilities from September 1^st^ 2005 until December 31^st^ 2012 formed the study population. Transfer in cases, patients with fewer than two CD4 tests and patients with follow-up for less than six months on HAART were excluded. The total sample size required was estimated for the combined accessible patients’ documents on HAART treatment in the two hospitals. Sample size calculations were done assuming random sampling for a finite population based on estimation of a 50% proportion, at a 5% significance level, 2% precision, and 15% estimated missing records. This put the sample size to be 1,304.

### Variables and Source of Data

The primary outcome variable was immunological failure as defined earlier. Predictor variables included adherence as measured by appointment for medication refill, baseline WHO clinical stage, baseline CD4 cell count, age, gender, and current tuberculosis. A document checklist was used to collect data from medical records and registers. The data collection checklist and data cleaning program codes were pretested prior to data collection and cleaning. This was done by a statistician among transferred-in patients found in one of the study facilities.

### Data Analysis

Epi Info version 3.5.1 statistical software (United States Centers for Disease Control and Prevention 2007) was used to clean the data while Stata version 12.0 was used to analyze it. Pattern of immunological monitoring was assessed using the national monitoring standard for determination of follow-up CD4 cell count for patients on antiretroviral therapy. The timing for evaluation of patients was every six months the first year and yearly then after [28]. A CD4 cell count test was said to have been done timely if it was done within one month of that particular follow-up month the test was supposed to be done. The expected number of follow-up CD4 cell count tests was calculated for those patients who were still on follow-up in that facility. A bar chart was used to show and contrast expected and actual CD4 cell count tests that were determined timely. Pattern of immunological response was evaluated at cohort level every six months. CD4 cell counts within two months of follow-up month of interest were used to determine CD4 cell count response at that month. A box plot was used to view this graphically. Chi square test was used to identify variables that have effect on pattern of immunological monitoring and immunological response after HAART initiation. Adequacy of immunological response was analyzed by taking as an outcome the percentage of those patients in whom the follow-up CD4 cell count achieved was more than or equal to 350. The median follow-up CD4 cell count was used as guide to decide what cut off to use. Time to the very first diagnosis of immunological treatment failure was studied using survival analysis. To identify predictors of immunological treatment failure, multivariable analysis using Cox proportional hazards regression analysis was used. Purposeful selection of variables was implemented as described by Hosmer and Lemeshow [29]. The final model was selected after checking for collinearity between covariates, the fulfillment of proportional hazards assumptions, and existence of extreme influential records.

The values of adherence and tuberculosis status varied with time. The variable ‘adherence’ was assigned a value of ‘0’ as long as patients were adherent to treatment with respect to their appointment to HAART medication prescription refill. If they failed to show up, this variable was assigned a value of ‘1’ for the time of follow-up after the time of interruption. This enabled the investigator to compare if the risk of immunological treatment failure varied between times after treatment interruption as compared to that before treatment interruption. In practical terms, this required splitting the observation time of patients into that up to the point at which treatment was discontinued, where the value to adherence was ‘0’, and the time period after that, where the value given to adherence was ‘1’. So, the time an individual spent being adherent to appointments was taken into account. This is justified for this study since patients who failed to show up for their medication refill in effect discontinued treatment for at least more than two weeks and thus fell beneath the 95% adherence limit, thus putting them at risk of experiencing treatment failure [30]. Tuberculosis was treated in the same way to assess if occurrence of incident tuberculosis after six months was a predictor of immunological failure. Such modeling of time-varying covariates required the use of extensions to the Cox proportional hazards regression modeling which was employed in this study. Lastly, since samples were taken from two different sites, accounting for within site correlation was made by stratification of hazard ratios by site. [29, 31–33]. P-value was set at 0.05. Datasets and analysis codes as well as instructions on how to use the datasets and analysis codes to obtain the same result as indicated below are presented as supporting information.

## Results

**Fig 1:**
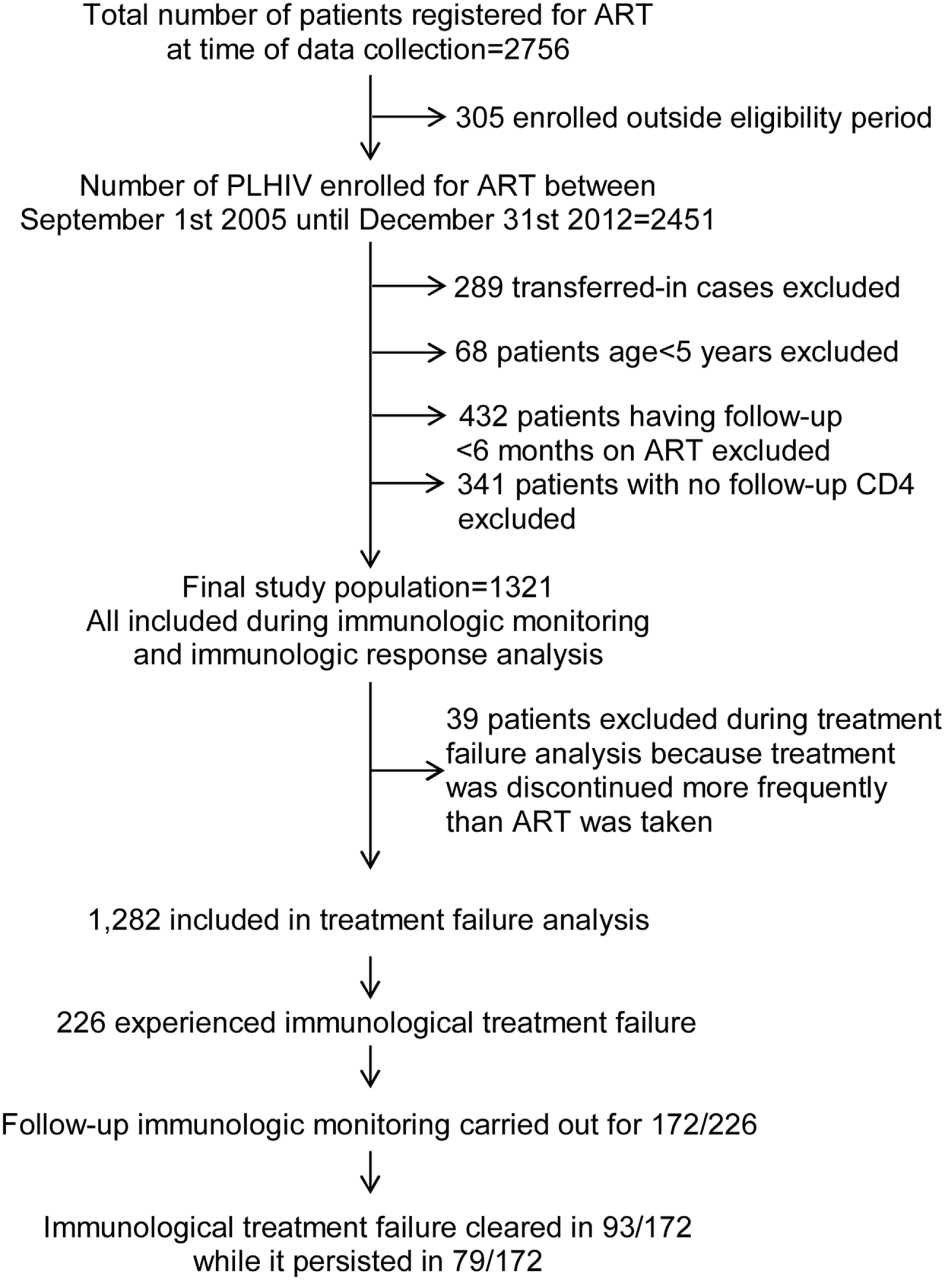
A schematic description of patient selection and follow-up. Of a possible 2,756 patients, 1,321 were eligible for inclusion in the study. 1,282 were eligible for inclusion in the final analysis for immunological treatment failure.

After applying eligibility criteria (Fig 1), there were a total of 1,321 documents of patients. Since the sample size calculated was 1,304, all were included in the analysis. The findings show that the median age of those in the study sample was 30 years (a range of 5–71 years). Females accounted for 63% (n = 831) of patients. Sixty per cent of patients (n = 786) were in WHO stage III or IV at HAART initiation. The median baseline CD4 cell count at HAART initiation was 156 cells per mm^3^ (a range of 1–1,221 cells/mm^3^). After the initiation of HAART, tuberculosis was diagnosed in 17.79% (n = 235) of patients. Eighteen per cent (n = 241) of patients showed poor adherence as they could not show up to their appointments for medication refill. These patients were followed up for a median time of 30 months (interquartile range: 15 to 48 months) after restarting treatment to censoring or diagnosis of failure. By the end of the follow-up, 73% (n = 960) of patients were still on HAART in the same facility where treatment was initiated. There was no difference with respect to being active on follow-up with regard to baseline covariates (age, gender, baseline WHO stage and baseline CD4 cell count). (Tables 1 and 2)

**Table 1:** Characteristics of patients on anti-retroviral therapy, (N = 1,321).

Variable	Category	Frequency, n	Per cent, %
**Age in years**	5–14	52	3.94
	>14	1269	96.06
**Gender**	Female	831	62.91
	Male	490	37.09
**WHO Stage**	I or II	535	40.50
	III or IV	786	59.50
Baseline CD4 cell count*	<100	378	28.61
	100–199	547	41.41
	200–349	359	27.18
	>349	37	2.80
**Tuberculosis**	No	1086	82.21
	Yes	235	17.79
**Adherence**	Good	1080	81.76
	Poor	241	18.24
**Final follow-up status**	Active	960	72.67
	Dead	44	3.33
	Drop	62	4.69
	Lost	4	0.30
	Stop	2	0.15
	Transferred out	249	18.85

* Measured in cells/mm^3^.

**Table 2:** Final follow-up status by demographic and baseline characteristics of study patients on anti-retroviral therapy (N = 1,321).

Variable	Category	Total, n	Number Active (%)	Pearson Chi Square	P- value
**Age in years**	5–14	52	38 (73.08)	0.0045	0.947
	>14	1269	922 (72.66)		
**Gender**	Female	831	599 (72.08)	0.3932	0.531
	Male	490	361 (73.67)		
**WHO Stage**	I or II	535	392 (73.27)	0.1623	0.687
	III or IV	786	568 (72.26)		
Baseline CD4 cell count*	<100	378	270 (71.43)	2.8564	0.414
	100–199	547	409 (74.77)		
	200–349	359	257 (71.59)		
	>349	37	24 (64.86)		

* Measured in cells/mm^3^.

### Pattern of Immunological Monitoring

**Fig 2:**
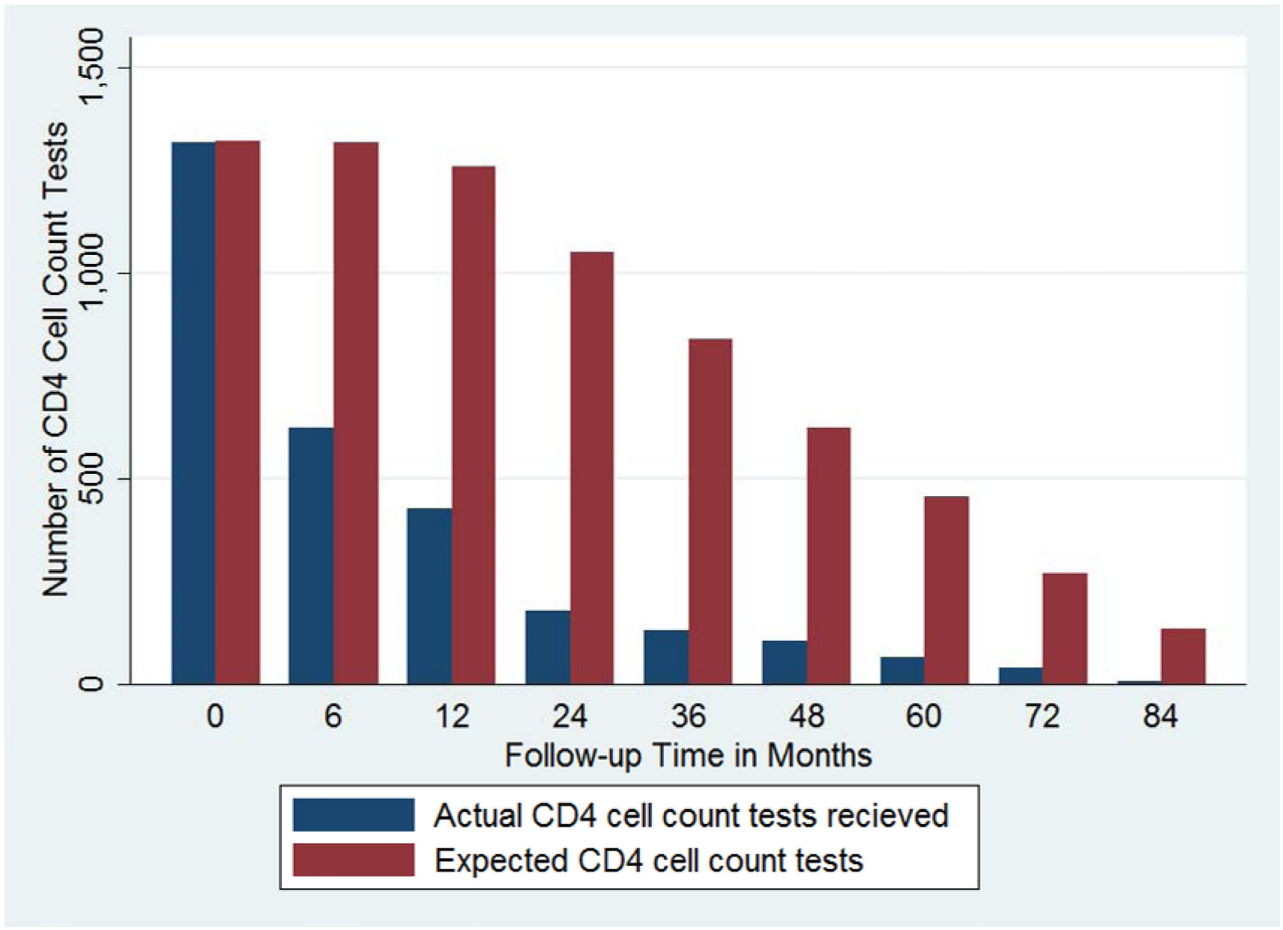
Pattern of CD4 cell count monitoring by follow-up time (N = 1,321). The actual number of CD4 cell count tests performed is seen to decline over time even taking into account the progressively lesser number of patients in care as the length of follow-up increased.

Taking into account only the time patients were on follow-up, the pattern of timely immunological monitoring was, in general, inadequate (Fig 2 and Table 3). It can be seen that all patients had baseline CD4 cell count testing at month zero. The number of CD4 cell count tests performed timely decreased to 46.31% (n = 610) at six months of follow-up while it was 33.04% (n = 417) at one year after ART initiation. It decreased and reached 7.35% (n = 10) for patients who were in follow-up for seven years. Overall, of the expected 7,285 baseline and follow-up CD4 cell count tests, only 2,885 tests were done in a timely fashion. This was 40.58% of the total expected, which is very low. There was no predilection for determination of follow-up CD4 cell count test in a timely fashion by age, gender, baseline WHO stage or baseline CD4 cell count (p value > 0.05 for all) (Table 4).

**Table 3:** Determination of timely follow-up CD4 testing by follow-up period in months among patients on anti-retroviral therapy (N = 1,321).

Follow-up Month	Expected number of CD4 tests	Number of follow-up CD4 tests provided	Per cent (%) CD4 tests provided
**0**	1321	1321	100.00
**6**	1317	610	46.31
**12**	1262	417	33.04
**24**	1053	179	17.00
**36**	842	132	15.68
**48**	628	108	17.20
**60**	459	68	14.81
**72**	271	40	14.76
**84**	136	10	7.35
**Total**	7289	2885	40.58

**Table 4:** Determination of timely follow-up CD4 cell count testing by demographic and baseline treatment variables among patients on anti-retroviral therapy (N = 1,321).

Variable	Category	Expected # of CD4 tests	Actual # of CD4 test performed	% Actual CD4 test performed	Chi square	P value
**Age in years**	5–14	278	100	35.97	1.5812	0.209
	>14	7012	2786	39.73		
**Gender**	Female	4578	1806	39.45	0.0993	0.753
	Male	2712	1080	39.82		
**WHO Stage**	I or II	2897	1150	39.70	0.0233	0.879
	III or IV	4393	1736	39.52		
Baseline CD4 cell count*	<100	2163	860	39.76	0.2358	0.972
	100–199	3089	1220	39.49		
	200–349	1843	726	39.39		
	>349	195	80	41.03		

* Measured in cells/mm^3^.

### Pattern of Immunological Response

**Fig 3:**
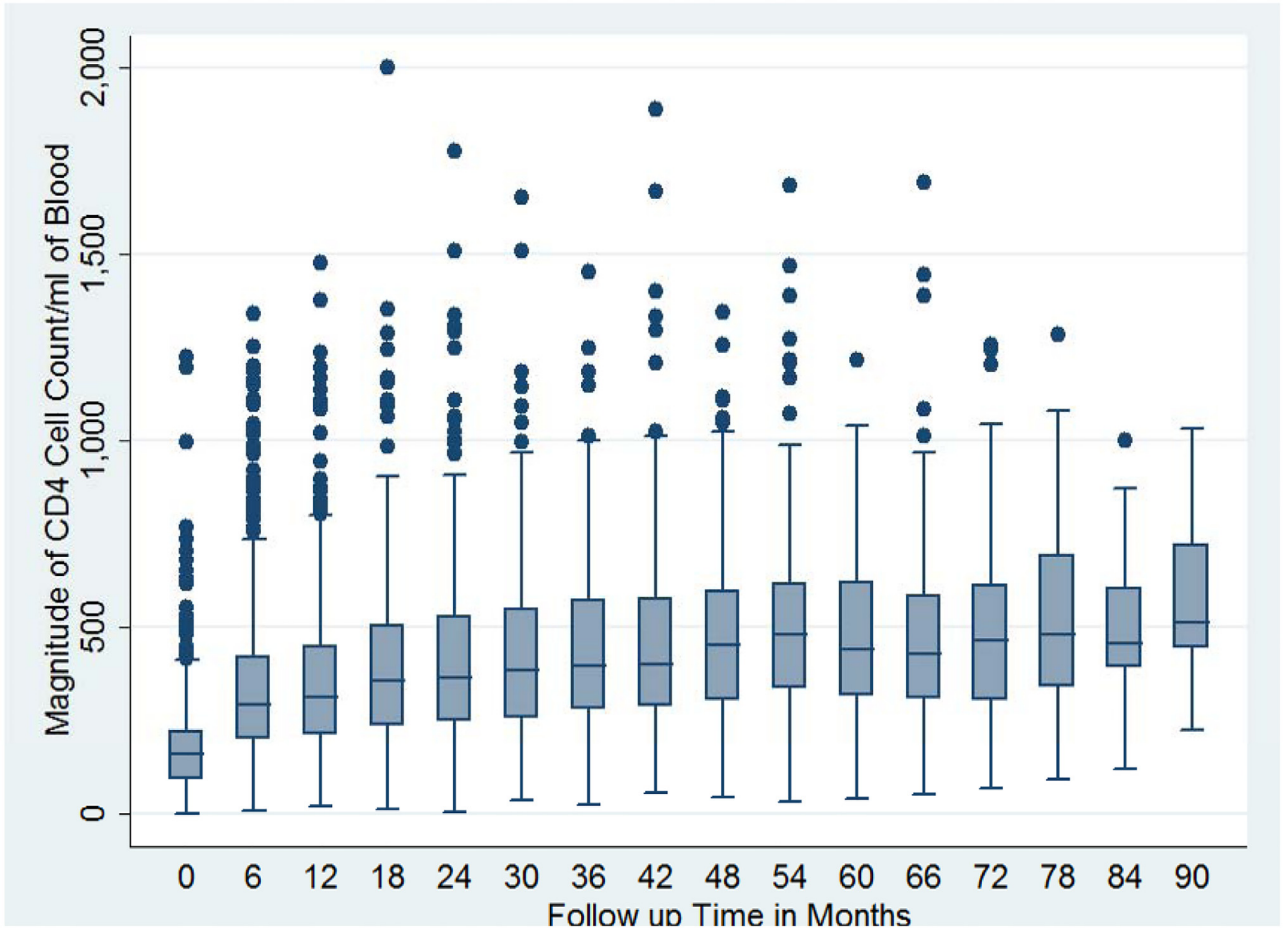
Box plot for magnitude of baseline and follow-up CD4 cell count among cohort of patients on anti-retroviral therapy by follow-up month (N = 1,321). The group level CD4 cell count as measured by median CD4 cell count at a particular follow-up time is seen to increase at first and plateau then after.

Immunological (CD4 cell count) recovery was observed among patients on antiretroviral therapy. The magnitude of follow-up CD4 cell count increase was rapid in the first few years of follow-up after starting antiretroviral therapy and reached a plateau thereafter (Fig 3). Children of less than 15 years of age, female patients, those who were at baseline WHO clinical stages I or II, as well as patients with a higher baseline CD4 cell count were able to achieve a higher proportion of follow-up CD4 cell count greater than or equal to 350 than their respective counterparts (p value < 0.05 for all) (Table 5).

**Table 5:** Magnitude of follow-up CD4 cell count by demographic and baseline treatment variables among patients on anti-retroviral therapy (N = 1,321).

Variable	Category	Total, n	Follow-up CD4 ≥350	% with follow-up CD4≥350	Chi square	P value
**Age in years**	5–14	165	146	88.48	78.9601	0.000
	>14	5076	2715	53.49		
**Gender**	Female	3330	2006	60.24	117.6648	0.000
	Male	1911	855	44.74		
**WHO Stage**	I or II	2079	1193	57.38	10.8558	0.001
	III or IV	3162	1668	52.75		
Baseline CD4 cell count*	<100	1569	512	32.63	568.0106	0.000
	100–199	2224	1253	56.34		
	200–349	1300	983	75.62		
	>349	148	113	76.35		

* Measured in cells/mm^3^.

### Immunological Treatment Failure

Of the 1,321 patients’ medical records reviewed, 2.95% (n = 39) discontinued their treatment for considerable time. Such patients would have needed additional follow-up before treatment failure diagnosis could be made, for which reason they were not evaluated for treatment failure. After excluding these patients, the prevalence of patients who experienced immunological failure at least once was 17.6% (n = 226) (95% confidence interval: 15.6%-19.8%). But, with further evaluation and follow-up CD4 cell count determination this percentage dropped to 11.5% (n = 147) (95% confidence interval: 9.8%-13.3%) (Table 6). Diagnosis of treatment failure was mostly based on the criteria ‘drop in follow-up CD4 cell count by 50% from follow-up peak’ (58%, n = 131) followed by the criteria ‘drop of follow-up CD4 cell count to or below baseline CD4 cell count’ (35%, n = 78) (Table 7). Having a follow-up immunological evaluation resulted in a decrease of the number of patients with immunological failure, with almost 46% of those with follow-up CD4 cell count coming out of it (Table 8). The percentage of those who came out of immunological treatment failure didn’t differ with immunological treatment failure diagnostic criteria, age, baseline WHO stage or CD4 cell while females were found to be marginally more likely to come out of it than males (p value = 0.05) as shown in supporting information. The overall percentage of patients on second line HAART was 1.67% (95% confidence interval 0.97%-2.36%). There were very few patients on second line HAART among those with confirmed immunological failure (7.53%, n = 7) (Table 9).

**Table 6:** Immunological treatment failure among patients on anti-retroviral therapy (N = 1,321).

Variable	Category	Frequency, n	Per cent,%
**Immunological failure**	No	1056	79.94
	Yes	226	17.11
	Not Evaluated	39	2.95
**Immunological failure (at last immunological evaluation)**	No	1135	85.92
	Yes	147	11.13
	Not Evaluated	39	2.95

**Table 7:** Diagnostic criteria for initial immunological failure among study patients on anti-retroviral therapy in (n = 226).

Diagnostic criteria	Frequency	Per cent
**Follow-up CD4 cell count dropped by at least 50% from peak value**	131	57.96
**CD4 cell count persistently below 100/mm** ^**3**^	17	7.52
**Follow-up CD4 cell count dropped to or below baseline**	78	34.51
**Total**	226	100.00

**Table 8:** Follow-up immunological evaluation and final immunological status of patients with immunological failure (n = 226).

Follow-up CD4 cell count testing	Immunological failure at last evaluation		Total, number (%*)
	Yes, number (%*)	No, number (%*)	
**No**	54 (100.00)	0 (0)	54 (100%)
**Yes**	93 (54.07)	79 (45.93)	172 (100%)
**Total**	147 (65.04)	79 (34.96)	226 (100%)

**Table 9:** Frequency distribution of patients on second line treatment among those with confirmed immunological failure (n = 93).

On second line HAART	Frequency, n	Per cent, %
**No**	86	92.47
**Yes**	7	7.53
**Total**	93	100.00

### Survival Analysis

**Fig 4:**
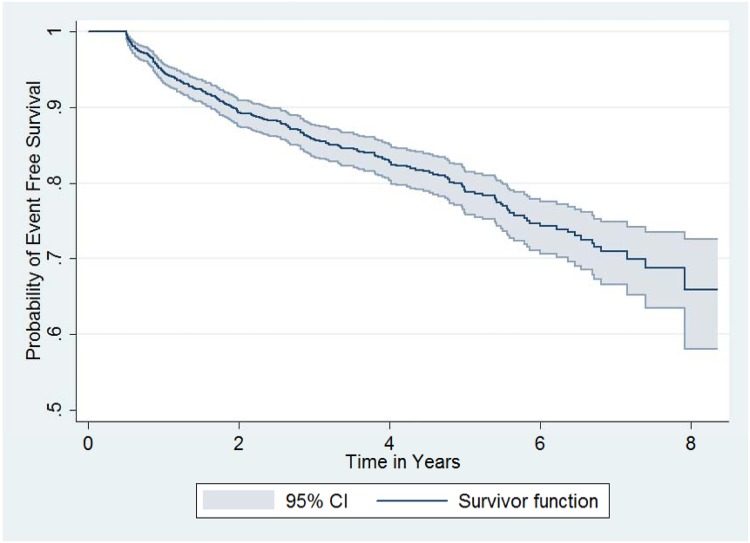
Kaplan-Meier survival functions for survival free of immunological treatment failure (n = 1,282). The even free survival probability, the event being free of immunological treatment failure, decreased steadily.

Patients not evaluated for treatment failure were excluded (n = 39) from survival analysis. This left 97% (n = 1,282) of patients. The median duration of follow-up from HAART initiation to diagnosis of immunological failure was 1.8 years (range: 0.51–7.92). For those who never experienced treatment failure, the median follow-up time was 3.73 years (0.51–8.36). The patient data scrutinized amounted to a total of 4,649 person-years at censoring or at diagnosis of immunological failure (Table 10). Table 11 and Fig 4 summarize event free survival, the event being the first instance of immunological treatment failure. It can be deduced that there had been a more or less steady drop in event free survival with time. At the end of the second year of follow-up, the probability of being free of immunological failure was 0.89 (95% CI: 0.87–0.91), while at end of five years it was 0.80 (95% CI: 0.77–0.82).

**Table 10:** Description of follow-up time in years for study patients on anti-retroviral therapy (n = 1,282).

	No. of subjects	Total time at risk	Mean	Minimum	Median	Maximum
**Failures**	226	543.08	2.40	0.51	1.80	7.92
**Censored**	1056	4091.23	3.87	0.51	3.72	8.36
**Total**	1282	4634.32	3.61	0.51	3.42	8.36

**Table 11:** Life table for immunological failure among patients on anti-retroviral therapy (n = 1,282).

Period	# at follow-up start	# with failure	Censored	Event free Survival	95% Confidence Interval
**0–1**	1282	67	52	0.95	0.93–0.96
**1–2**	1163	60	184	0.89	0.87–0.91
**2–3**	919	33	170	0.86	0.84–0.88
**3–4**	716	21	181	0.83	0.80–0.85
**4–5**	514	18	143	0.80	0.77–0.82
**5–6**	353	16	144	0.75	0.72–0.78
**6–7**	193	8	92	0.71	0.67–0.75
**7–8**	93	3	71	0.67	0.61–0.73

**Fig 5:**
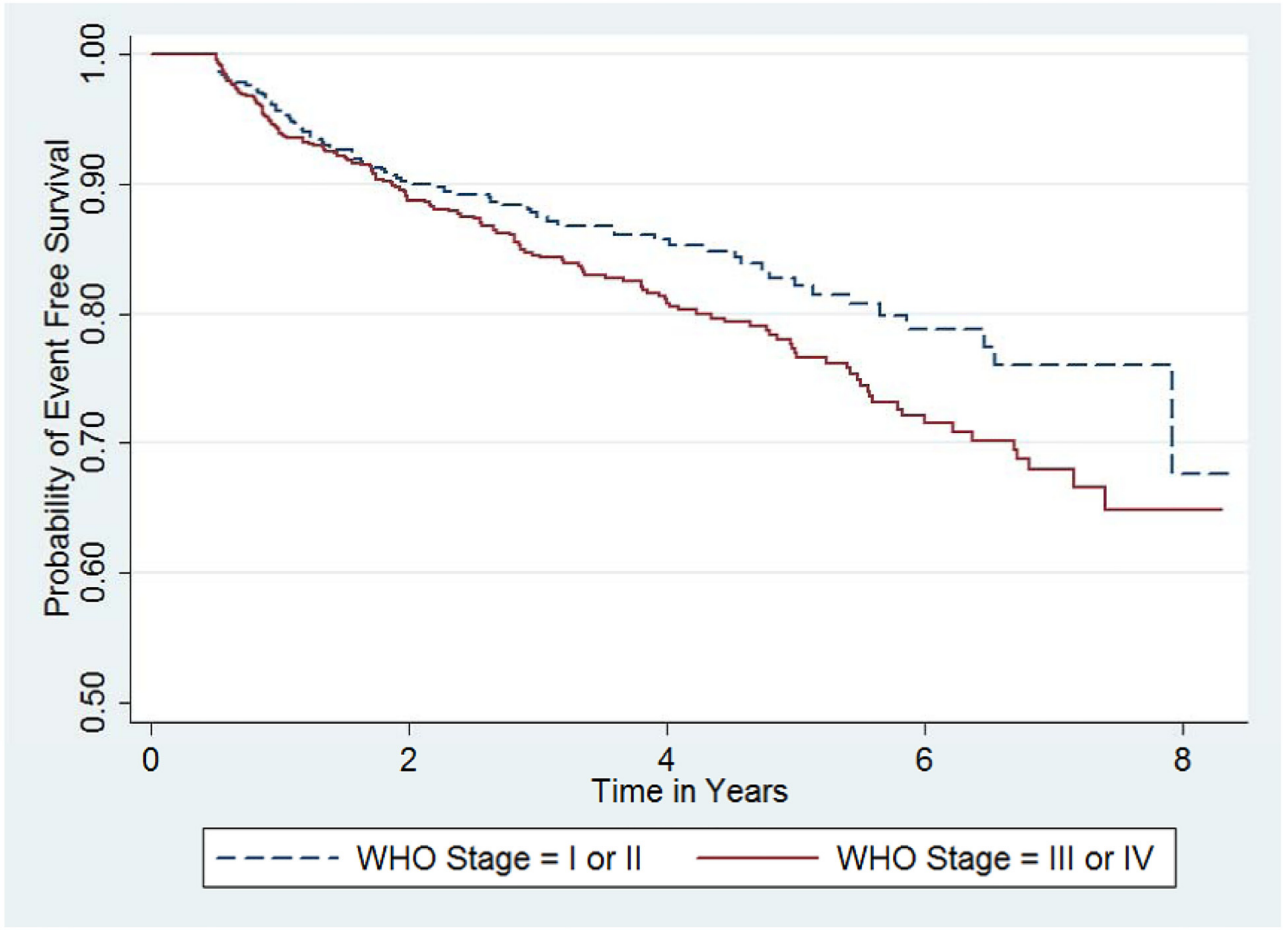
Kaplan-Meier survival functions by WHO Stage (n = 1,282). Event free survival, the even being free of immunological treatment failure, was higher for patients with WHO stages I or II as compared to those with WHO stage III or IV.

**Fig 6:**
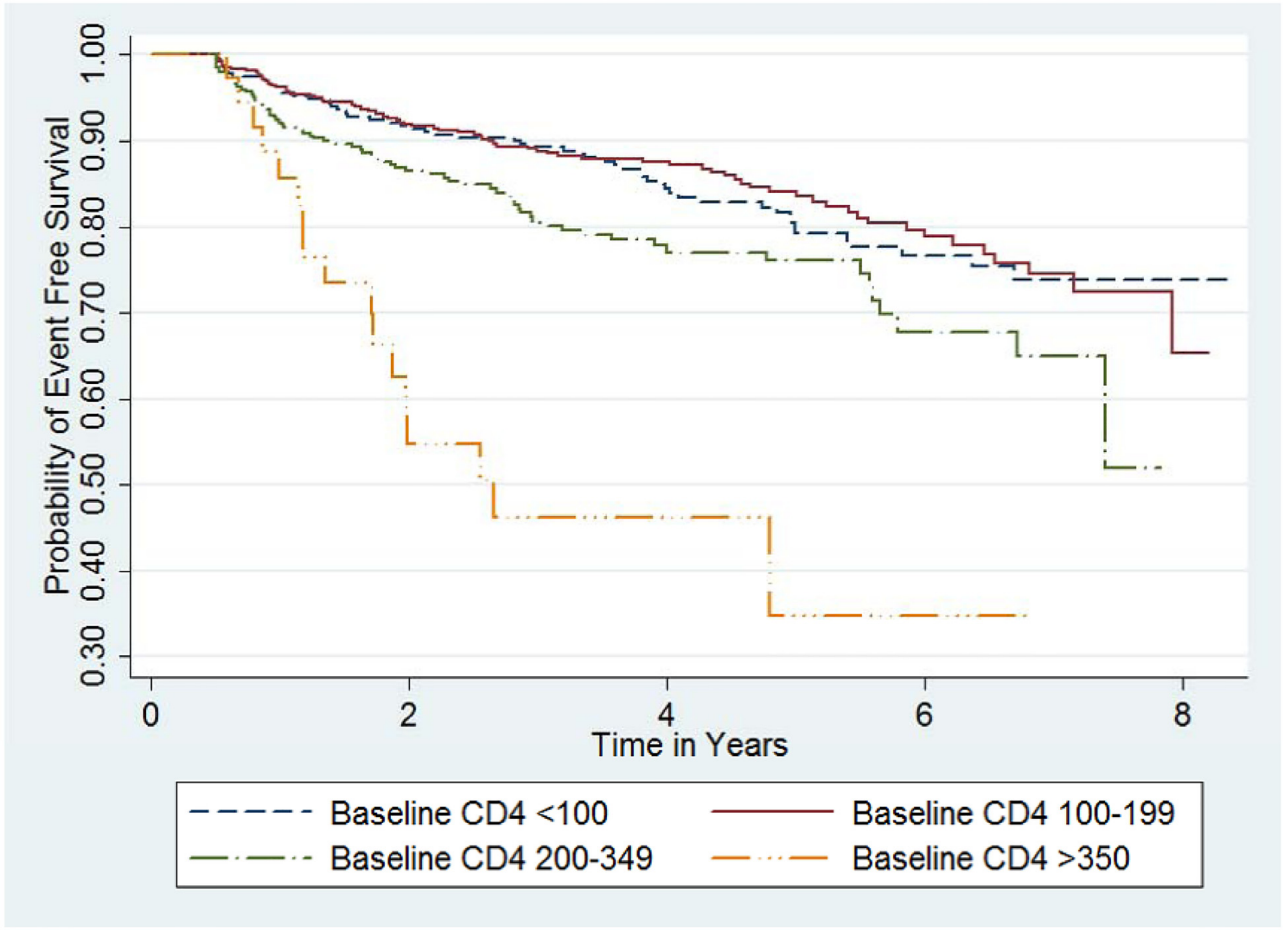
Kaplan-Meier survival functions by baseline CD4 cell count group (n = 1,282). Event free survival, the even being free of immunological treatment failure, was lower for patients with baseline CD4 cell count more than 349 as compared to those with other CD4 cell count categories.

During univariable as well as multivariable analysis, being at WHO Stage III or IV or having a higher CD4 cell count at baseline were associated with being at level of increased hazard for immunological failure (Figs 5 and 6). Patients at WHO Stage III or IV had a hazard function which is 33% higher than the reference (adjusted hazard rate ratio of 2.718^(0.2840678)^ = 1.33). While an increase in baseline CD4 cell count by 100 cells/mm^3^ increased the hazard of immunological failure by 34% (adjusted hazard rate ratio of 2.718^(100x0.0029061)^ = 1.34). None of the other covariates (age, gender, tuberculosis, adherence to picking up medication refill on time) predicted immunological treatment failure. (Table 12)

**Table 12:** Cox proportional hazards regression analysis for predictors of immunological failure among patients on anti-retroviral therapy (n = 1,282).

	Unadjusted		adjusted	
Covariate	Coefficient	P value	Coefficient	P value
**Age**	0.0058875	0.405		
**Gender**	0.1542597	0.255		
**WHO Stage**	**0.2956083**	**0.037**	0.2840678**	**0.045**
**Baseline CD4 cell count (cells/mm** ^**3**^ **)**	**0.0029745**	**0.000**	0.0029061***	**0.000**
**Tuberculosis**	-0.0218439	0.970		
Adherence*	0.1445758	0.451		

* Adherence to medication refill.

** Equivalent to hazard ratio of 1.33.

*** Equivalent to hazard ratio of 1.34 per 100 increase of CD4 cell count.

## Discussion

Despite the adequacy of group level immunological recovery, immunological failure was observed at an individual level. The proportion of patients who ever experienced immunological failure was 17.6% (95% confidence interval: 15.6%-19.8%). However with further evaluation and follow-up CD4 cell count determination, it dropped to 11.5%. Since a significant number of patients who were re-evaluated after immunological failure didn’t fulfil immunological failure criteria, it could be possible that the actual percentage of patients with immunological failure would have been even lower than this, had there been proper follow-up immunological evaluation.

This is the first paper that assessed the level of immunological failure for both adult and paediatric patients on HAART according to the WHO 2010 criteria for diagnosing immunological treatment failure [17] and the first study that reported the effect of follow-up immunological evaluation on the level of immunological failure in Ethiopia. Various studies have reported on the prevalence of immunological failure. One study in northern Ethiopia reported a prevalence of 22% (n = 89) among adults, which was higher than the finding from the present study [23]. Immunological failure at last evaluation was similar to two studies carried out in the Ethiopian capital Addis Ababa (8.2%, n = 79) and western Ethiopia (11.5%, n = 11) [21, 22]; direct comparison is not possible, though, since both studies focused on paediatric patients with a mean age of six years, and follow-up duration was shorter in the second study (mean follow-up duration of 13.7 months). In other sub-Saharan African countries, studies point to an overall higher prevalence of immunological failure, at a range of 10%-32%, even when the follow-up duration was shorter compared to that of this study. [5, 6, 34, 35] In a study in Nigeria, the prevalence of unconfirmed immunological failure was 32% and dropped to 10% after diagnosis was confirmed with subsequent CD4 cell count tests [34].

It should be noted that the gold standard for determining antiretroviral treatment failure is viral load testing, which is not routinely available in Ethiopia. For this reason, interpretation and utilization of immunological failure criteria should be based on its strength and limitations. This is provided by the predictive values of immunological failure on virologic failure. Several studies have made that assessment and found out that the benefit of screening patients with the WHO’s immunological failure criteria is in ruling out virologic failure among those who don’t have immunological failure according to those criteria. This is because the WHO’s immunological failure criteria has very high negative predictive value (87% to 99% depending on the study) but low positive predictive value (9% to 37% depending on the study) [34–36]. For example, in this study it means that one is 87% to 99% sure that 82% of the study patients who were found not to have immunological failure were likely to be free of virological failure. On the other hand, of the 18% who were found to have immunological failure, we are certain that only 9% to 37% have virological failure, which is very low. As a result of this limitation of immunologic failure criteria, the WHO recommends the use of targeted viral load testing for confirming antiretroviral treatment failure for those who fulfil immunological failure criteria in resource limited settings where routine virologiclal monitoring isn’t available. [30]

The overall prevalence of patients on second line HAART, 1.67% (95% confidence interval 0.97%-2.36%), is comparable to what was reported by Assefa et al [24], which in their study was 2.13% (95% confidence interval: 1.8% to 2.5%) among 7,451 patients at the second year of HAART. However, in the current study, a lot more patients should have been initiated on second line HAART since there were more patients with confirmed immunological failure. The consequence of unmanaged treatment failure could be increased risk of developing resistant viral strains leading to a reduced drug choice for second line therapy, as well as increased morbidity and mortality [30]. Thus, it should be looked into why those patients were not managed as such. A number of factors could contribute for this: providers’ failure to identify treatment failure; lack of knowledge, or confidence to act; lack of adequate second line drugs, etc. [17]. For some patients, maintaining their first line regimen was justified since after subsequent tests they came out of immunological failure. According to the WHO, interventions including adherence assessment and addressing gaps for identified barriers could result in improvement of immunological outcomes. If these interventions were followed, it would be expected that some of those who were in failure could come out of it [30].

The fact that the majority with failure (58%) were diagnosed with the criteria ‘50% drop from peak follow-up CD4 cell count value’ implies that they experienced an initial increase in CD4 cell count before it slumped down later on. This is consistent with the finding that the majority in follow-up experience considerable CD4 cell count recovery especially in the first year of follow-up. This trend of adequate initial recovery has been reported in other studies as well [4, 19, 20, 37]. Being paediatric, female, in advanced WHO stage at HAART initiation and having higher baseline CD4 cell count were associated with reaching a higher CD4 cell count. These findings are supported by one study in Uganda [38]. In a study done in eastern Ethiopia, there was no such association evident between the variables stated above and CD4 cell count recovery [20]. The findings were different, however, probably because different outcome parameters were used in the form of CD4 cell count increment in the above mentioned study in contrast to achieving a follow-up CD4 cell count of greater than or equal to 350 used in the current study. In a more recent study in northern Ethiopia that used a CD4 cell count of 200 as cut off amongst 100 adults and 100 children, no association was observed as well [37].

The suboptimal level of immunological monitoring, which could be one contributing cause for some patients to remain in treatment failure, has been documented in another study in southern Ethiopia [18]. This is similar in other sub-Saharan African countries as well. A study in Malawi, for instance, showed that only 5,361 (30.2%) ART patients out of 17,737 on follow-up received follow-up CD4 cell count testing. And out of 15,924 patients who were eligible for two tests, only 1,006 (6.3%) received testing as per guideline [39]. The reasons for poor follow-up CD4 cell count testing could be multifactorial: providers not requesting tests, patients not coming for testing, lack of awareness from the patients’ side, or a breakdown of machines or lack of reagents at facilities [40, 41].

Two factors were important in predicting immunological treatment failure. Baseline WHO stage and baseline CD4 cell count. Those with higher baseline WHO stages (III or IV) or higher baseline CD4 cell count were found to be at high risk for immunological treatment failure. Other variables like age, gender, adherence to medication refill, and tuberculosis were not associated with it. There are studies that assessed predictors of immunological failure but comparison was not possible with most because different immunological failure criteria were used. For instance, Kassa et al used the following criteria to define immunological failure: increase of CD4 cell count by fewer than 50 cells/μl at month six, and the criteria of less than 100 cells/μl at months 18 and 24 of HAART; Anude et al used the criteria ‘fall of follow-up CD4 cell count to baseline or below’, or ‘an increase in the CD4 cell count of less than 50 cells/mm^3^ at one year’ after HAART initiation. All these criteria are different from that issued by the WHO [19, 30, 42] and some of the criterion they used were rather measures of adequate recovery than failure. There are two studies that consistently used the WHO criteria to define immunological failure: one was carried out in Soweto, South Africa [5] while the other in Mozambique. In the South African study, level of baseline CD4 cell count was not found to be associated with immunological failure. This may be because of the low sample size in that study (n = 456). Findings of no association were similar with respect to age, gender, tuberculosis, and treatment interruption. In the Mozambique study, the CD4 cell count association was similar to findings form the current study [43]. The fact that there was no association between adherence to antiretroviral medication refill and immunological treatment failure was a surprising finding. This is despite adequate follow-up after restarting treatment for those who discontinued treatment. This could be because patients who were “adherent” to medication refills may not be taking their medication properly [30]. After all, picking up medications doesn’t say much about proper intake. Achieving adherence ≥95% for taking HAART medications depends on a number of factors like family support, disclosure, availability of reminders, and presence of opportunistic infections, pill burden, and so on [44]. Therefore, even though adherence to medication refill is a reliable means of identifying those with adherence problems, it should be complemented with other adherence assessment measures like pill count and self-report otherwise it could underestimate the true burden of poor adherence to treatment, bearing in mind that these alternative measures may be less reliable because of social desirability response bias [7, 30, 45–47].

This study may have important inputs both for clinical practice and policy design on the issue of immunological monitoring and management of those with immunological failure while on HAART. Firstly, the cut off CD4 cell count for initiating HAART for adults and adolescents in Ethiopia has been raised in Ethiopia to be 500 since 2013. In addition to that mothers are initiating lifelong HAART at any CD4 level for PMTCT purposes. The same is true for HIV infected children and adolescents below 14 years of age who are all eligible for HAART in spite of WHO staging or baseline CD4 cell count [48]. This means that an increasing number of patients may be starting treatment at higher baseline CD4 cell count level which, according to the findings of this study, may increase the number of patients with immunological failure. Secondly, the availability and effective utilization of CD4 cell count testing machines including point of care tests should be improved since follow-up CD4 cell count testing alone reduced the prevalence of failure by a third, thus, reducing unnecessary switching of regimen [27]. Thirdly, the level of those on second line HAART treatment and those with immunological failure was not comparable indicating that health care providers may need additional support in deciding treatment plans. This emphasizes the need for implementing better immunological evaluation mechanisms. This could be in the form of utilizing more sensitive measures of treatment failure like viral load testing. Existing treatment follow-up tools used in following patients in routine clinical care should be improved to make it easier for health care providers to manage patients. A tool that can be used to address this is shown in Table 13. It comes with simple criteria to screen patients in order to identify those with increased risk of immunological treatment failure. These criteria are having CD4 cell count above 350 and having baseline WHO stage III or IV. The 350 cut-off was chosen in order to simplify the screening criteria and it is based on the Kaplan-Meir survival curve shown in Fig 6 that shows that the event free survival was relatively low for those with that cut-off. Therefore, patients fulfilling one of those criteria may be prioritized in case of resource limitations for monitoring. In addition to that, the tool highlights follow-up periods when immunological monitoring needs to be carried out and prompts decision as to whether immunological failure is present or not. And in case there is immunological failure, it prompts further actions that need to be taken.

**Table 13:** Immunologic failure risk assessment and management tool.

Circle any that applies: 1. Baseline CD4 cell count ≥350/mm^3^? Yes No 2. Baseline WHO Stage III or IV? Yes No							
High risk for immunologic treatment failure if answer to either of the questions is Yes. Low risk otherwise.							
Date	Month on ART	Adherence*	ART regimen	CD4 count	Immunologic failure** (Y or N)	Decision***	Appointment date
	0						
	1						
	2						
	3						
	4						
	5						
	6						
	7						
	8						
	9						
	10						
	11						
	12						
	13						
	14						
	15						
	16						
	17						
	18						
	19						
	20						
	21						
	22						
	23						
	24						

*Adherence = G (Good) if missed doses<3 per month; F (Fair) if missed doses 3–5 and 3–9 for 30 and 60 doses regimen per month respectively; P (Poor) if missed doses >5 or >9 for 30 and 60 doses regimen per month respectively.

**Immunological failure criteria: 1.50% below maximum follow-up CD4 cell count; 2. at or below baseline CD4 cell count; 3. persistently below 200. The presence of any one of these criteria in patients who have been taking antiretroviral treatment for at least six months and is still on treatment is used to diagnose it [17, 27].

*** Decision: (Use if there is immunologic failure)

0 = Re-evaluate with CD4 cell count

1 = Re-enforce adherence

2 = Re-evaluate with viral load

3 = Regimen change to second line

Despite the overall positive contributions of this study for clinical practice and program implementation, it has important limitations. Since this was a retrospective study based on existing medical records, the effect of possibly erroneous entries as well as incomplete entries of data should not be underestimated. For instance, these reasons have led us to drop the variable weight. In addition, the study could not assess the effect of clinical disease progression on immunological failure except for tuberculosis as follow-up staging (treatment staging) was not well documented. Not all patients were active on follow-up at last observation because of treatment interruption or transfer to other facility for follow-up. For this reason, immunological failure could only be measured for as long as patients were on follow-up. This effect was, however, accounted for during survival analysis, which is specifically, designed to analyze such data. Moreover, there was no association between baseline covariates and being active on follow-up at last observation making the effect of incomplete observations minimal. Also, since only two hospitals were selected for convenience, the overall prevalence of immunological failure couldn’t be generalized for the whole region.

This study merely identified the problem of poor immunological determination. Therefore, further studies need be made to address barriers to proper CD4 cell count determination. In the face of confirmed immunological failure, very few patients were managed by shifting antiretroviral treatment regimen to second line according to the guideline. Hence, providers’ knowledge, attitude, and practice to managing patients with immunological failure should be evaluated in order to design an intervention to address gaps in care being observed. Also, the proposed tool to monitor and manage immunological assessment and management for those with failure needs to be validated and piloted before implementation.

## Conclusion

Immunological treatment failure was prevalent in the study setting. There is suboptimal level of immunological monitoring which if addressed may contribute to reduction in the number of patients with immunologic failure diagnosis, and thus unnecessary switching to second line therapy where targeted viral load testing is not yet possible. Patients with confirmed immunological failure diagnosis were not managed as per the guideline which may contribute for further drug resistance. Further studies need to be made to identify root causes of poor monitoring and management of patients with failure.

## Supporting Information

S1 Dataset Immunologic monitoring dataset. (DTA)

S2 Dataset Immunologic response dataset. (DTA)

S3 Dataset Immunologic failure dataset. (DTA)

S1 Dofile Immunologic monitoring dofile. (DO)

S2 Dofile Immunologic response dofile. (DO)

S3 Dofile Immunologic failure dofile. (DO)

S1 Table Immunological failure diagnostic criteria and immunological failure after follow-up immunological evaluation (n = 172). (DOCX)

S2 Table Baseline CD4 cell count and immunological failure after follow-up immunological evaluation (n = 172). (DOCX)

S1 Text Instruction indicating how to use datasets and dofiles to reach at results. (DOCX)
