# Supplementary material for: Prevalence and Predictors of Immunological Failure among HIV Patients on HAART in Southern Ethiopia
Source: PLoS One. 2015 May 11;10(5):e0125826. doi: 10.1371/journal.pone.0125826 (PMC4427446; doi:10.1371/journal.pone.0125826)
Supplement: S2 Table — (DOCX) [file pone.0125826.s008.docx]

S2 Table Immunological failure diagnostic criteria and immunological failure after follow-up immunological evaluation (n=172)

| **Diagnostic criteria^*^** | **# with treatment failure re-evaluated** | **# came out of treatment failure** | **% came out of treatment failure** | **Chi square** | **P value** |
| --- | --- | --- | --- | --- | --- |
| 1 | 99 | 43 | 43.43 | 2.78 | 0.25 |
| 2 | 13 | 4 | 30.77 |  |  |
| 3 | 60 | 32 | 53.33 |  |  |

**^*^** 1=Follow-up CD4 cell count dropped by at least 50% from peak value

2=CD4 cell count persistently below 100/mm^3^

3=Follow-up CD4 cell count dropped to or below baseline

S3 Table Baseline cd4 cell count and immunological failure after follow-up immunological evaluation (n=172)

| **Variable** | **Category** | **# with treatment failure re-evaluated** | **% came out of treatment failure** | **Chi square** | **P value** |
| --- | --- | --- | --- | --- | --- |
| Age | 5-14 | 9 | 33.33 | 0.61 | 0.44 |
|  | >14 | 163 | 46.63 |  |  |
| Gender | Female | 104 | 51.92 | 3.80 | **0.05** |
|  | Male | 68 | 36.76 |  |  |
| Baseline WHO Stage | I or II | 61 | 45.90 | 0.00 | 0.99 |
|  | III or IV | 111 | 45.95 |  |  |
| Baseline CD4 cell count* | <100 | 48 | 41.67 | 3.06 | 0.38 |
|  | 100-199 | 58 | 53.45 |  |  |
|  | 200-349 | 50 | 46.00 |  |  |
|  | >349 | 16 | 31.25 |  |  |

* Measured in cells/mm^3^
