# Supplementary material for: Prevalence and Predictors of Immunological Failure among HIV Patients on HAART in Southern Ethiopia
Source: PLoS One. 2015 May 11;10(5):e0125826. doi: 10.1371/journal.pone.0125826 (PMC4427446; doi:10.1371/journal.pone.0125826)
Supplement: S1 Text — (DOCX) [file pone.0125826.s009.docx]

Dear reader,

Please follow the instructions below in order to come up with outputs as shown in the manuscript.

Step 1: Put the three datasets and dofiles on drive ‘D:\’ of your of your computer. The data sets relate to the three objectives of the study: immunologic monitoring, immunologic response, and immunologic failure.

Step 2: Open your Stata, click ‘File’, click ‘Do’, and double click on the do file you want to run to get the desired output.

Step 3: Print the output if you want to view it later on.

N.B: Once you run S3_Dofile.do, the dataset S3_Dataset.dat changes and saving it will keep the changes made during the analysis. Running it while these changes have been made will not be possible as a result of the changes made. Thus you have to replace it with the original dataset.

Cheers!
